# PP82 Environmental Sustainability Assessment Across The Medical Device Life Cycle: Taking The Viewpoint Of Low Resource Settings

**DOI:** 10.1017/S0266462325102997

**Published:** 2025-12-29

**Authors:** Debjani Mueller, Melissa Pegg, Abhirup Dutta Majumdar

## Abstract

**Introduction:**

Globally, healthcare services contribute five percent of total greenhouse gas (GHG) emissions. Advances in biomedical research have led to innovative technologies, but the use of fossil fuel reliant products is a critical environmental issue. The burden is even greater in low resource settings (LRS) with increasing waste management issues. Real world studies can shed light on the environmental footprint of health care and help prioritize assessments.

**Methods:**

Various policies and guidance are being developed on environmental sustainability of healthcare. However, it is imperative that health technology at different levels of care is studied empirically, taking a holistic approach to the environmental impact from a planetary health perspective. By presenting research from LRS, we can understand how use of devices and services have different environmental consequences depending on established regulatory frameworks. We conducted a rapid review with snowballing to understand how LRS have adapted to unmet needs and tried to solve these using a circular economy approach.

**Results:**

The footprint of a typical cataract operation in a high resource setting was 182 kg of carbon dioxide equivalent. Efficient systems and equipment reuse reduces this to 6 kg in a LRS, a 30-fold reduction. Despite both having positive clinical outcomes, one phacoemulsification surgery in the UK emits 20 times more GHG than the equivalent in India. Resource scarcity necessitated circular economy practices like “repair” and “reuse” to keep products in circulation. An electrosurgical unit, typically lacking in LRS, is designed to be durable, repairable, upgradable, and reusable even during electrical failures. Planetary and human health across all settings require resource optimization, innovative materials engineering, and reusable solutions.

**Conclusions:**

Urgent research is needed to understand the potential impacts of more sustainable health technology innovation across eye care in different settings. A broader range of environmental impacts beyond GHG emissions studied across the life cycle of devices is necessary. Routine inclusion of environmental outcomes in clinical studies and explicit identification, testing, and promotion of sustainable practices will help promote sustainable decisions.

